# *Penaeus monodon* fibrinogen-related lectin interacts with lipopolysaccharide and β-1,3-glucan binding protein to activate the innate immune system

**DOI:** 10.1042/BCJ20253314

**Published:** 2025-12-17

**Authors:** Patcharin Wilasluck, Pongsakorn Sukonthamarn, Anchalee Tassanakajon, Kittikhun Wangkanont

**Affiliations:** 1 Center of Excellence for Molecular Biology and Genomics of Shrimp, Department of Biochemistry, Faculty of Science, Chulalongkorn University, Bangkok, 10330, Thailand; 2 Center of Excellence in Molecular Crop, Department of Biochemistry, Faculty of Science, Chulalongkorn University, Bangkok, 10330, Thailand

**Keywords:** coiled-coil protein, fibrinogen-related lectin, innate immunity, lipopolysaccharide and β-1,3-glucan binding protein, *Penaeus monodon*

## Abstract

The black tiger shrimp, *Penaeus monodon*, is cultivated commercially in many countries, including Thailand. However, diseases are major limiting factors in shrimp production. Understanding the shrimp immune responses to pathogens will be essential for health management and disease control. Fibrinogen-related proteins (FREPs) act as pattern recognition receptors by recognizing carbohydrate residues on pathogen surfaces. Here, we report that *P. monodon* FREP (*Pm*FREP) interacts with the *P. monodon* lipopolysaccharide and β-1,3-glucan binding protein (*Pm*LGBP) to co-ordinate the innate immune response in shrimp. Like *Pm*LGBP, *Pm*FREP participates in the prophenoloxidase (proPO)-activating cascade upon the *Vibrio parahaemolyticus* acute hepatopancreatic necrosis disease-causing strain infection. *Pm*FREP knockdown results in a significant decrease in phenoloxidase (PO) activity, whereas recombinant *Pm*FREP injection noticeably increases the enzyme activity. The coiled-coil (CC) region at the N-terminus of *Pm*FREP mediates the direct binding between *Pm*FREP and *Pm*LGBP to enhance PO activity. Our results suggest that *Pm*FREP recognizes the pathogens to form an immunological complex with *Pm*LGBP through the CC region, subsequently enhancing the proPO-activating cascade to eliminate the pathogens. Therefore, *Pm*FREP serves as a key component for signaling transduction in the shrimp immune defense.

## Introduction

Black tiger shrimp, *Penaeus monodon*, is an economic aquatic animal cultivated to supply global food demand. However, key limiting factors of shrimp aquaculture are infectious diseases. Major pathogens that cause severe losses to shrimp aquaculture [1] especially white spot syndrome virus [2], yellow head virus [3], *Enterocytozoon hepatopenaei* [4], and *Vibrio parahaemolyticus* acute hepatopancreatic necrosis disease (AHPND)-causing strain (*VP*_AHPND_) [5]. To overcome these diseases, understanding the mechanism of the shrimp immunity against pathogens is necessary. Although there have been numerous studies on shrimp immunity, unlike the mammalian system, many pathways remain poorly documented. Shrimp rely on their innate immune system to defend against pathogen infections. The immune responses are initiated through the binding between the pattern recognition receptors (PRRs) and the pathogen-associated molecular patterns (PAMPs) on the surface of pathogens. PRRs play an important function in recognizing non-self molecules. The interaction between PRRs and PAMPs activates signaling pathways, which in turn activate the immunological defense mechanism [6]. PRRs of shrimp are classified into numerous categories, such as Toll-like receptors [7,8], C-type lectins (CTLs) [9], lipopolysaccharide and β-1,3-glucan binding proteins (LGBPs) [10], scavenger receptors [11,12], and thioester-containing proteins (TEPs) [13]. Fibrinogen-related proteins (FREPs) are also PRRs that are lectins or carbohydrate-binding proteins. These lectins have a fibrinogen-like (FBG) domain as a carbohydrate recognition domain. Whereas FREPs are typically categorized as lectins, certain variants do not necessarily exhibit carbohydrate-binding properties. FREPs can interact with acetylated molecules [14], lipoteichoic acid [15], protein or peptide such as fibrinogen γC [16,17], fibrinogen γ'C [18] and tenascin-C [19]. In invertebrates, FREPs play an essential part in recognizing pathogens in innate immunity by distinguishing between the carbohydrate structures on the host cells versus the invading cells [20]. In addition to their function as PRRs, FREPs also facilitate bacterial agglutination [21], encapsulation [22], and activation of immune effector mechanisms, such as the expression of antimicrobial peptides [23]. FREPs were discovered in several shrimp species such as *Penaeus vannamei* [24–26], *Fenneropenaeus merguiensis* [27], *Marsupenaeus japonicus* [28], and *Macrobrachium rosenbergii* [29].

In *P. monodon*, three FREP-like genes [Penlectin5 (PL5)-1, PL5-2, and PL5-3] were investigated [30]. Here, we are focusing on PL5-1 or *Pm*FREP. This protein was first purified from the shrimp hemolymph [31]. *Pm*FREP can bind peptidoglycan, N-acetyl sugar, and bacteria, including *Pseudomonas aeruginosa* and *V. parahaemolyticus,* in a Ca^2+^-independent manner [32]. After *VP*_AHPND_ infection, there was an increase in the expression of *PmFREP* in the stomach [33].

*Pm*FREP comprises two coiled-coil (CC) regions at the N-terminus and a FBG domain that functions as a carbohydrate recognition domain (CRD) at the C-terminus. Because of the CC regions, *Pm*FREP assembles into a higher-ordered structure [32]. The CC region of lectins plays a significant function in structural stability, ligand binding, oligomerization, and protein–protein interaction. The CC regions of human lung surfactant protein D mediate the assembly of an oligomeric structure [34]. Human mannose-binding protein (MBP) trimerizes through the CC region into a tetranectin-like structure [35,36]. This oligomerization is required for its ability to recognize and bind to pathogens. Moreover, the CC domain of the CTL from the kuruma shrimp, *M. japonicus*, could bind to the domeless receptor and activate the Janus kinase/signal transducer and activator of transcription (JAK/STAT) pathway against bacteria [37]. It is currently established that *Pm*FREP can bind the pathogen; however, the signaling mechanisms that are involved after binding remain unidentified. This research aims to elucidate the signaling pathway that is triggered after pathogen binding to *Pm*FREP. Here, we hypothesize that *Pm*FREP might interact with other immune proteins to form an immunological complex, subsequently activating the immune signaling cascade. We also theorize that the CC region of *Pm*FREP is likely to be necessary in this binding process. Understanding the immunological role of *Pm*FREP could not only enhance the knowledge of shrimp immunity but would also open avenues for developing novel disease control strategies in shrimp aquaculture. Moreover, the study of *Pm*FREP will contribute to a broader understanding of lectin functions in animals.

## Results

### Expression pattern of *PmFREP* in hemocytes after *VP*_AHPND_ infection

**Figure 1:**
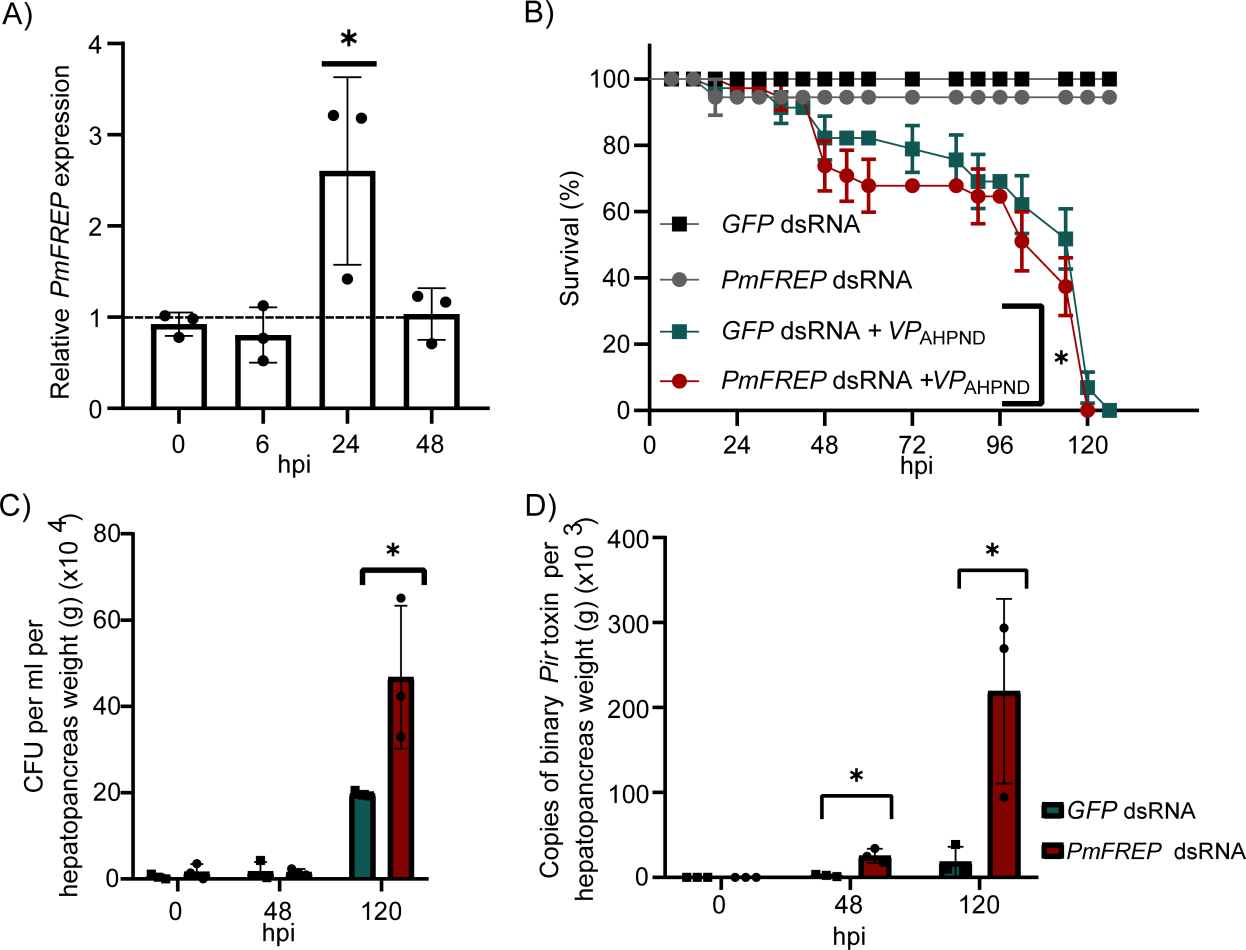
*PmFREP* expression and the effect of *PmFREP* suppression during *VP*_AHPND_ infection. **(A**) *PmFREP* gene expression levels in the hemocytes at 0, 6, 24, and 48 hpi. (**B**) The effect of *PmFREP* suppression on the survival rate. (**C**) Bacteria CFU in the hepatopancreas. (**D**) Binary *Pir* toxin gene copy number in the hepatopancreas. The data were analyzed by t-test. Asterisks indicate statistically significant differences with a *P*-value of less than 0.05. CFU, colony-forming units.

To examine the participation of *Pm*FREP during *VP*_AHPND_ infection, *PmFREP* transcripts in hemocytes were quantified at 0, 6, 24, and 48 hours post-infection (hpi) with *VP*_AHPND_. The transcripts were normalized using *P. monodon* elongation factor 1-α *(PmEF1α)* as an internal control and compared with normal shrimp as a control group. The *PmFREP* transcripts were up-regulated by approximately 2.6-fold at 24 hpi (Figure 1A). This result suggested that *PmFREP* might be involved in shrimp immune response against the *VP*_AHPND_ infection. Therefore, we further investigate the effect of *PmFREP* suppression during *VP*_AHPND_ infection.

### Effect of *PmFREP* suppression on survival rate and bacterial counts after *VP*_AHPND_ infection

To study the role of *Pm*FREP in shrimp immune response, the *PmFREP* gene was suppressed using RNA interference (RNAi). *GFP* double-stranded RNA (dsRNA)-injected shrimp served as a control group, and the *PmEF1α* transcript was used as an internal control. At 48 hours after dsRNA injection, shrimp were immersed in 5 × 10^5^ cells/ml *VP*_AHPND_. We successfully suppressed *PmFREP* gene expression at 48 hours by injection with 5 µg dsRNA/g shrimp (Supplementary Figure S1). *PmFREP* suppression resulted in a significantly lower survival rate compared with *GFP* dsRNA-injected shrimp (Figure 1B). The survival rate of *PmFREP* knockdown shrimp dropped to 0% within 120 hpi, while the *GFP* dsRNA group dropped to 0% at 126 hpi. *PmFREP* knockdown shrimp without infection can survive throughout the experiment. The colony-forming units (CFU) from the hepatopancreas of *PmFREP* knockdown shrimp were higher than the *GFP* dsRNA group by approximately 2.4-fold at 120 hpi (Figure 1C). To confirm the infection, *binary pir toxin* gene copy number was determined in the hepatopancreas. The toxin transcripts in *PmFREP* knockdown shrimp were higher than *GFP* dsRNA-injected shrimp by approximately 8.0-fold and 8.6-fold at 48 and 120 hpi, respectively (Figure 1D). This result indicated that *Pm*FREP plays a significant role in shrimp immunity against the *VP*_AHPND_ infection.

### Effects of *PmFREP* suppression on other immune gene expressions

**Figure 2:**
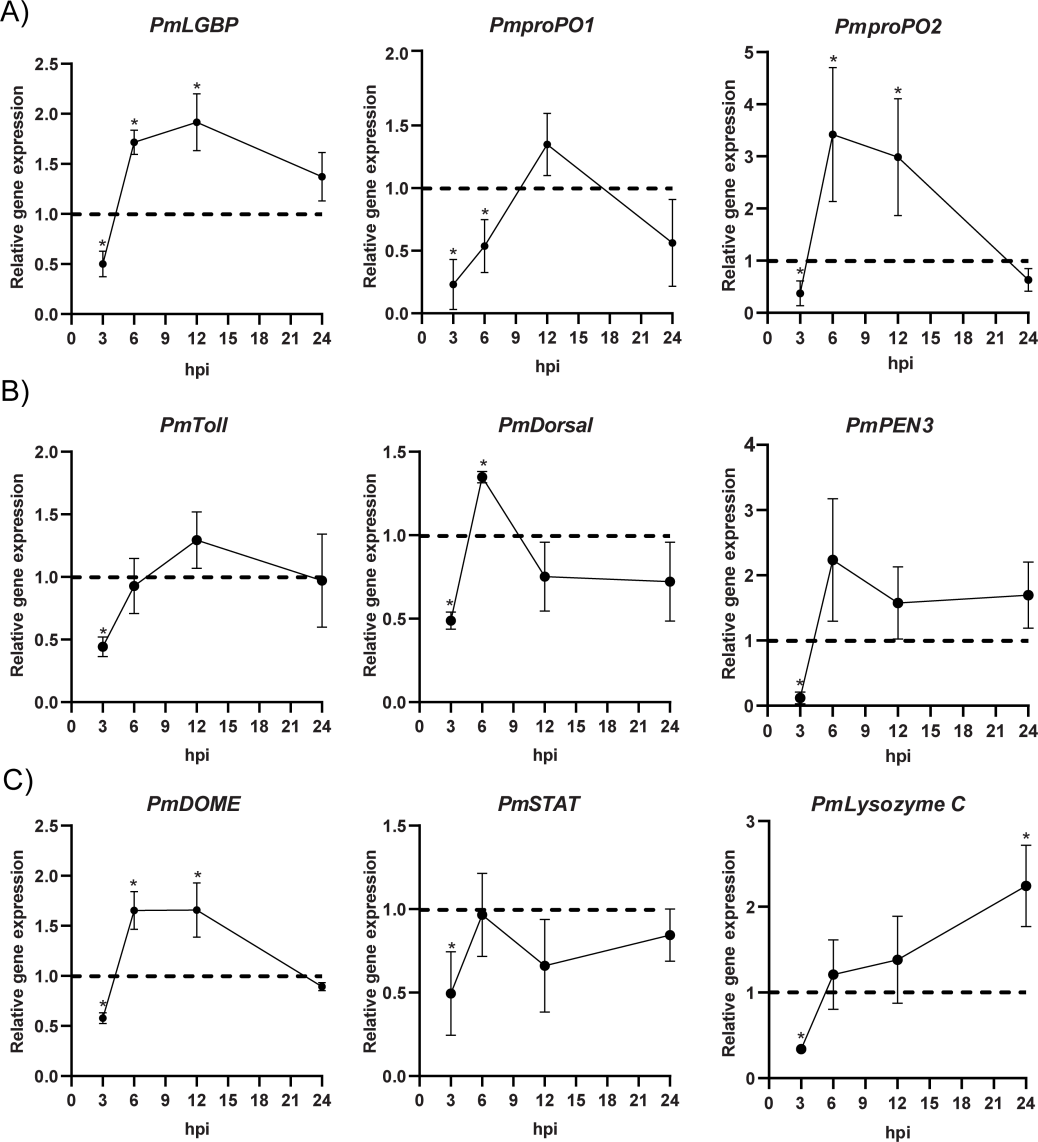
Effect of *PmFREP* suppression on immune gene expressions determined by qRT-PCR of (**A**) the proPO-associated genes, (**B**) Toll pathway-related genes, and (**C**) JAK/STAT pathway-related genes during *VP*_AHPND_ infection. *GFP* dsRNA-injected shrimp served as the control group to normalize each data set. The *EF1α* was used as an internal control. The data were analyzed by t-test. Each point represents average relative expressions of three independent replicates ± 1 SD (error bars). Significant differences compared with the control were shown as an asterisk (*p* < 0.05). *EF1α*, elongation factor 1α; SD, standard deviation.

To confirm the function of *Pm*FREP in shrimp immune response during *VP*_AHPND_ infection, the effects of *PmFREP* suppression on the expression of immune-related genes were investigated. The *PmFREP* gene was suppressed, followed by *VP*_AHPND_ infection, and the quantitative real-time PCR (qRT-PCR) was carried out. The shrimp injected with *GFP* dsRNA served as the control group. This research focused on the effect of *PmFREP* suppression on three associated immunological pathways, including the proPO pathway (*PmLGBP*, *PmproPO 1,* and *PmproPO 2*), the Toll pathway (*PmToll*, *PmDorsal,* and *PmPEN 3*), and the JAK/STAT pathway (*PmDOME*, *PmSTAT,* and *PmLysozyme C*). For the proPO-associated gene expression (Figure 2A), *PmLGBP* was down-regulated at 3 hpi (0.5-fold), subsequently up-regulated at 6 hpi and 12 hpi (1.7- and 1.9-fold, respectively), before getting back to the normal level at 24 hpi. *PmproPO 1 and PmproPO 2* showed down-regulation at 3 hpi (0.2- and 0.4-fold, respectively). At 6 hpi, *PmproPO 1* was still down-regulated (0.6-fold) whereas *PmproPO 2* showed up-regulation (3.4-fold). *PmproPO 1* returned to normal expression at 12 hpi, whereas *PmproPO 2* still increased (2.9-fold) before returning to normal level at 24 hpi. For the Toll pathway (Figure 2B), *PmToll* and *PmPEN 3* were down-regulated at 3 hpi (0.4- and 0.1-fold, respectively) and returned to normal levels at 6 hpi. *PmDorsal* was down-regulated at 3 hpi (0.5-fold), increased at 6 hpi (1.3-fold), and dropped to normal level at 12 hpi. For the JAK/STAT pathway (Figure 2C), *PmDOME*, *PmSTAT,* and *PmLysozyme C* were decreased at 3 hpi (0.6-, 0.5-, and 0.3-fold, respectively). At 6 hpi and 12 hpi, *PmDOME* was up-regulated (1.7-fold) and dropped to normal expression at 24 hpi, while *PmSTAT* and *PmLysozyme C* were returned to normal expression at 6 hpi. However, *PmLysozyme C* was up-regulated at 24 hpi approximately 2.2-fold. The *PmFREP* knockdown resulted in a delay of the immune system signals in shrimp, indicating that *Pm*FREP might play a crucial role in immune signal transduction after *VP*_AHPND_ binding.

### *Pm*FREP binding partner identification by a pull-down assay

**Figure 3:**
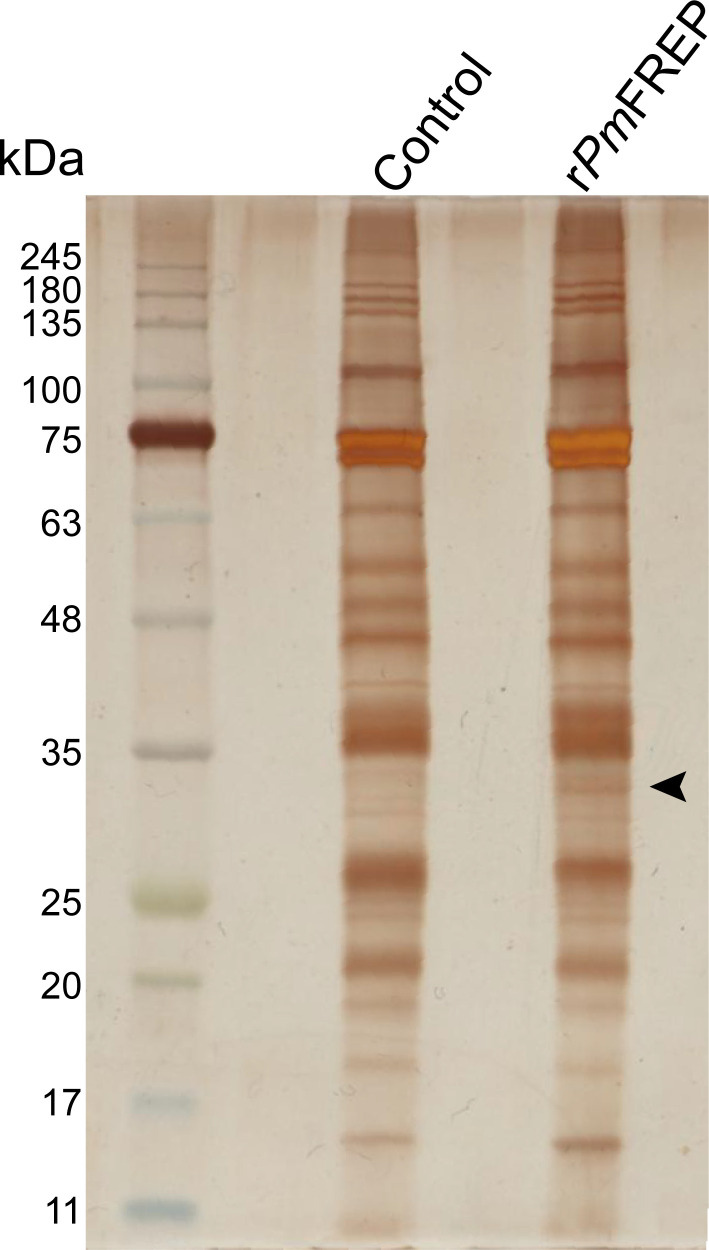
Silver-stained SDS-PAGE analysis of the *Pm*FREP pull-down assay against the hemolymph of *VP*_AHPND_-infected shrimp. The band that was chosen for mass spectrometric analysis is indicated with an arrow.

The mechanism by which *Pm*FREP activates the immunological signaling cascade after pathogen binding remains unclear. To narrow down the immune signaling pathway associated with *Pm*FREP, the pull-down experiment was performed to identify *Pm*FREP binding partners. The insect cell expression system was employed to produce r*Pm*FREP, which was subsequently purified using nickel-nitrilotriacetic acid (Ni-NTA) column chromatography [32] (Supplementary Figure S2A). The protein was immobilized onto *N*-hydroxysuccinimide (NHS)-activated magnetic beads and used to pull down the *Pm*FREP binding partner from the hemolymph of infected shrimp. The quenched beads served as a control experiment (Figure 3). A band in the elution fraction from the r*Pm*FREP-immobilized beads (lane r*Pm*FREP) that differed from the control experiment was observed. This band was cut and identified using LC-MS/MS. Table 1 presents the candidates of *Pm*FREP binding partners. Better coverage of the *Pm*LGBP (6.83) compared with other proteins suggested that *Pm*LGBP was likely a binding partner of *Pm*FREP. The apparent molecular weight of the band observed also agreed with that of *Pm*LGBP, which was known to migrate around 31 kDa instead of the expected 41.5 kDa [38].

**Table 1:** *Pm*FREP binding partner candidate proteins determined by LC-MS/MS

GenBank Accession Number	Description	Coverage	Protein Length (Amino Acids)	Molecular Weight (kDa)	Isoelectric Point (pI)
AEX08659	Lipopolysaccharide and β-1,3-glucan binding protein	6.83	366	41.5	4.67
Q9U572	Hemolymph clottable protein	3.83	1670	188.3	5.49
A1KYZ2	Tropomyosin	3.17	284	32.8	4.75
P83944	Alanine racemase	5.83	120	13.3	8.31

### Demonstration of *Pm*FREP and *Pm*LGBP binding by a curdlan binding assay

**Figure 4:**
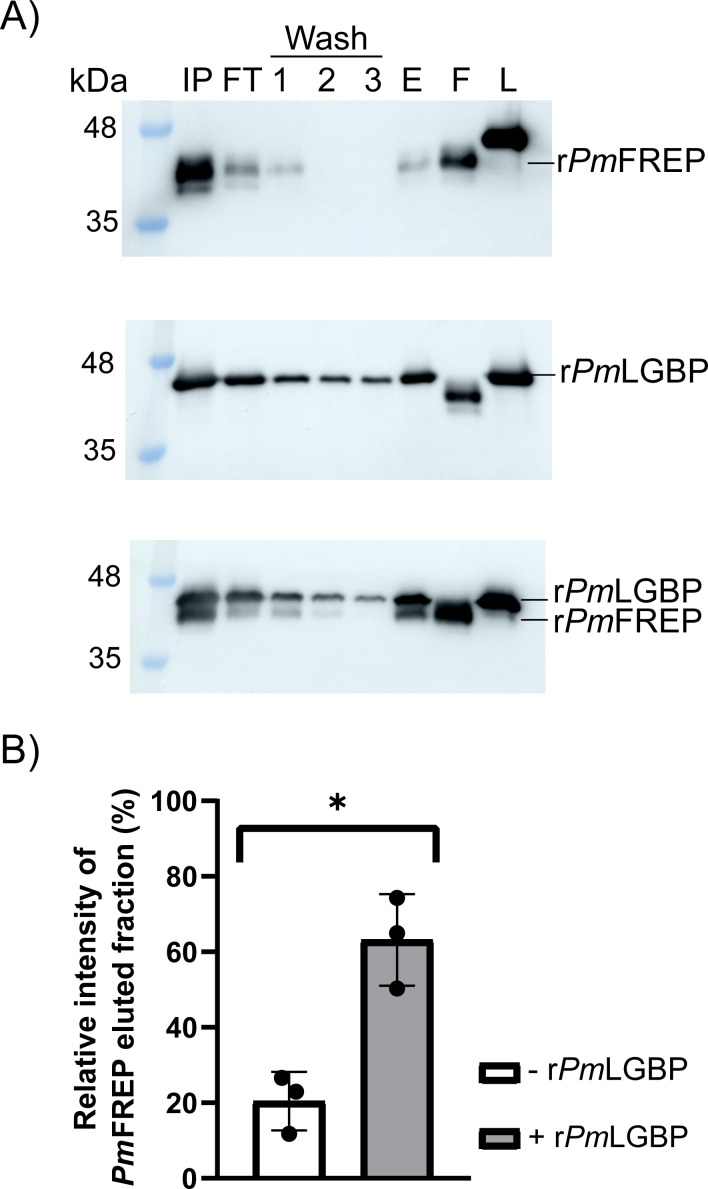
Interaction between r*Pm*FREP and r*Pm*LGBP using the curdlan binding assay. **(A**) Western blot analysis of the curdlan binding assay. IP=input, FT=flow-through, Wash=three sequential wash fractions, E=elution fraction, F=r*Pm*FREP, and L=r*Pm*LGBP. The top panel is the control experiment with only r*Pm*FREP. The middle panel is the control experiment with only r*Pm*LGBP. The bottom panel is the experiment with both r*Pm*FREP and r*Pm*LGBP. (**B**) Densitometry of the r*Pm*FREP in the elution fraction (triplicate, two other replicates in Supplementary Figure S3). The data were analyzed by t-test. A significant difference compared with control was shown as an asterisk (*p* < 0.05).

To validate the binding of *Pm*FREP and *Pm*LGBP, a curdlan binding assay was conducted. The recombinant *Pm*LGBP (r*Pm*LGBP) was successfully expressed using an insect cell expression system and purified by Ni-NTA affinity chromatography (Supplementary Figure S2 E). The curdlan binding experiment was carried out by incubating r*Pm*FREP and r*Pm*LGBP with curdlan. The incubation of r*Pm*FREP with curdlan or r*Pm*LGBP with curdlan served as a control experiment. The results suggested that r*Pm*FREP can bind weakly with curdlan (Figure 4A), and % intensity of the eluted fraction is approximately 20.5% (Figure 4B), while r*Pm*LGBP had a strong interaction with curdlan. When both r*Pm*FREP and r*Pm*LGBP were incubated with curdlan, more *Pm*FREP was eluted in an elution fraction compared with the control experiment, approximately 3.1-fold. This result demonstrated that r*Pm*FREP directly interacted with r*Pm*LGBP, suggesting that they form an immunological complex for subsequent biological function.

### Effect of *PmFREP* and *PmLGBP* suppression on PO activity

**Figure 5:**
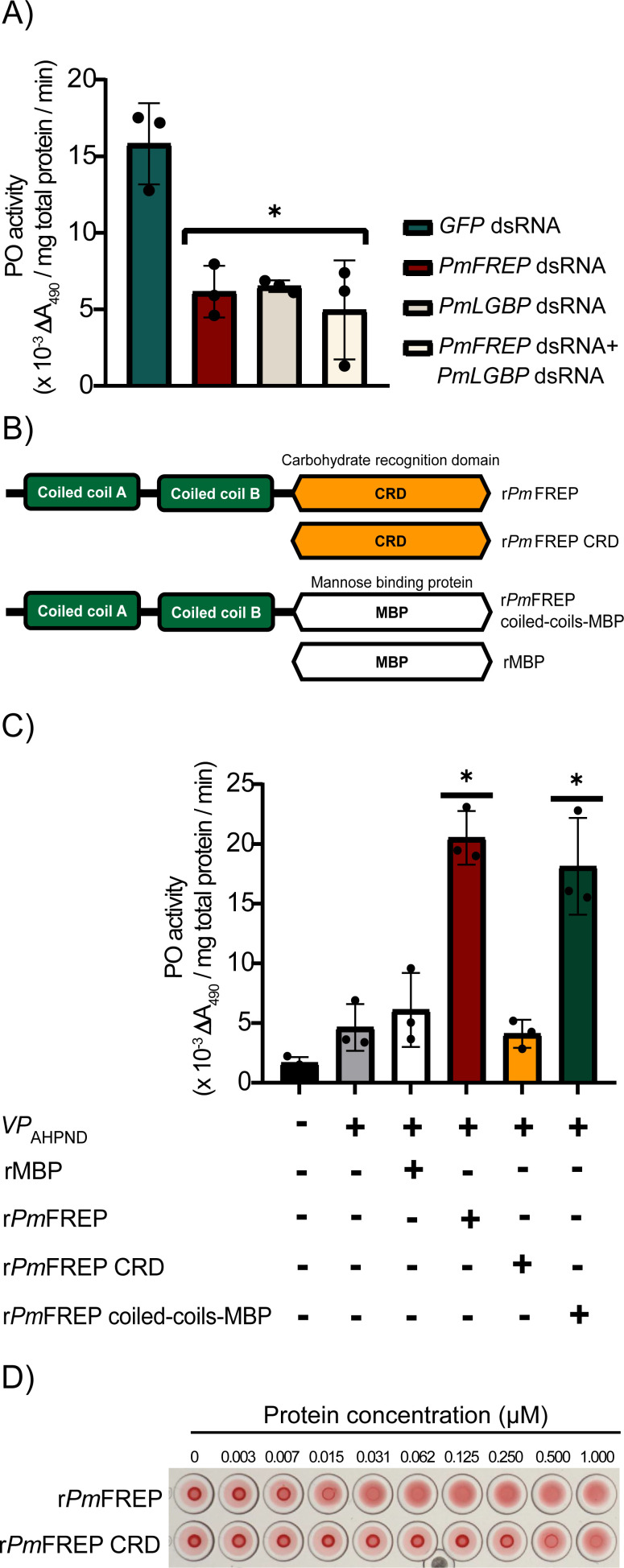
The effect of *PmFREP* suppression or r*Pm*FREP injection on PO activity. **(A**) The effect of *PmFREP* and *PmLGBP* suppression on PO activity. The *GFP* dsRNA-injected shrimp served as a control group. (**B**) Domain diagrams of *Pm*FREP and r*Pm*FREP coiled-coil (CC) constructs. (**C**) PO activity of r*PmFREP-*injected shrimp and r*Pm*FREP CC-MBP*-*injected shrimp. The rMBP injection served as a control. (**D**) Verification of r*Pm*FREP and r*Pm*FREP CRD biological activity by porcine red blood cell agglutination. The data were analyzed by one-way analysis of variance (ANOVA). Each bar in the graph represents the mean ± 1 SD (error bars) of three independently replicated studies with an asterisk indicating a statistically significant difference at *p* < 0.05. rMBP, recombinant mannose-binding protein; SD, standard deviation.

*Pm*LGBP is a receptor in the proPO pathway and could bind with *Pm*FREP. Thus, it is likely that *Pm*FREP is participating in the proPO pathway as well. To observe the involvement of *PmFREP* in the proPO pathway, *PmFREP* was suppressed by using the RNAi technique, and *GFP* dsRNA-injected shrimp were used as the control group. At 48 hours after *PmFREP* suppression, shrimp were infected with *VP*_AHPND_ by immersion. The phenoloxidase (PO) activity was determined after 24 hpi. The *PmFREP*-knockdown shrimp and *PmLGBP*-knockdown shrimp showed lower PO activity than the control group, approximately 2.6- and 2.5-fold, respectively (Figure 5A). Although double knockdown of *PmFREP* and *PmLGBP* dsRNA-injected shrimp showed PO activity less than the control group by ∼ 3.2-fold, PO activity does not differ significantly with single knockdown experiments. This study suggested that *PmFREP* plays a part in the proPO activation cascade.

### Enhancement of PO activity by r*Pm*FREP

To confirm the participation of *Pm*FREP in the proPO activation cascade, shrimp were injected with r*Pm*FREP constructs (Figure 5B)*,* and at 24 hours after injection, shrimp were immersed in *VP*_AHPND_. Injection of shrimp with r*Pm*FREP resulted in higher PO activity than control shrimp, approximately 3.4-fold (Figure 5C). Further study was performed to investigate which domain of *Pm*FREP could activate PO activity by injecting r*Pm*FREP CRD or r*Pm*FREP CC-MBP into shrimp. The r*Pm*FREP CRD was produced by an insect cell expression system (Supplementary Figure S2B). We successfully expressed r*Pm*FREP CC-MBP in the periplasmic space of *Escherichia coli* Tuner (DE3) (Supplementary Figure S2C). For the control experiment, recombinant mannose-binding protein (rMBP) was injected into shrimp. The PO activity of r*Pm*FREP CRD-injected shrimp showed no difference with control shrimp, while the PO activity of r*Pm*FREP CC-MBP-injected shrimp increased approximately 3.0-fold of PO activity. The PO activity in shrimp was enhanced by r*Pm*FREP or r*Pm*FREP CC-MBP. Therefore, *Pm*LGBP binding to *Pm*FREP involved the CC regions at the N-terminus of *Pm*FREP.

### The r*Pm*FREP CRD activity

To verify that r*Pm*FREP CRD was functional and that the inability of r*Pm*FREP CRD to activate the proPO activation cascade was not due to incorrect folding of r*Pm*FREP CRD, the hemagglutination and the N-acetylglucosamine (GlcNAc) column binding assays were performed. The r*Pm*FREP CRD agglutinated porcine red blood cells with the protein concentration of at least 0.5 µM. However, it is less effective than r*Pm*FREP, which requires a minimum protein concentration of 0.015 µM for agglutination (Figure 5D). This was likely due to the lack of the oligomeric structure of the r*Pm*FREP CRD. Nevertheless, r*Pm*FREP CRD was functional in the hemagglutination assay. Moreover, the GlcNAc binding assay revealed that r*Pm*FREP and r*Pm*FREP CRD were bound to the GlcNAc column and were eluted with 100 mM GlcNAc. In contrast, recombinant *Leptospira interrogans* dihydrofolate reductase-like enzyme (r*Li*DHFRL) [39], a non-binding control, did not bind the GlcNAc column and came out in the wash fractions (Supplementary Figure S4). These results indicated that r*Pm*FREP CRD was correctly folded and functional but failed to stimulate the PO activity.

### Binding affinity of *Pm*FREP and *Pm*LGBP

**Figure 6:**
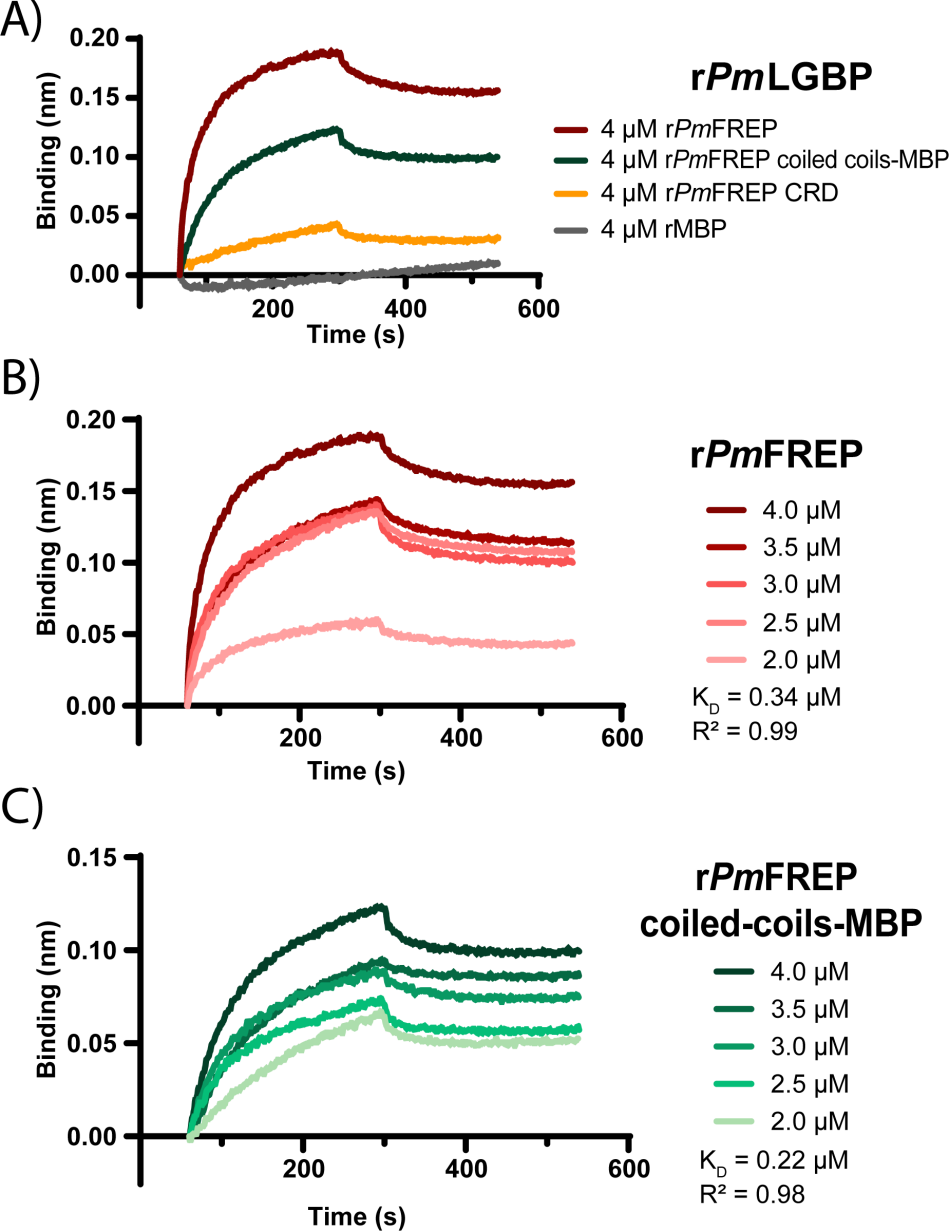
Binding of *Pm*LGBP to r*Pm*FREP by biolayer interferometry (BLI). The probe was immobilized with r*Pm*LGBP. **(A**) Various r*Pm*FREP constructs were the analytes. rMBP served as a control. (**B**) Binding curves of r*Pm*LGBP and various concentrations of r*Pm*FREP. (**C**) Binding curves of r*Pm*LGBP and various concentrations of r*Pm*FREP CC-MBP.

To confirm that *Pm*FREP interacts with *Pm*LGBP via CC regions at the N-terminus of *Pm*FREP and to measure the binding affinity, biolayer interferometry (BLI) was employed. The r*Pm*LGBP was immobilized on the biosensor. Each domain of *Pm*FREP served as an analyte, and rMBP served as a control. The results revealed that just the r*Pm*FREP and r*Pm*FREP CC-MBP gave robust binding curves with good fit (R^2^ > 0.95), and showed higher binding signals compared with r*Pm*FREP CRD and rMBP (Figure 6A). We further determined the dissociation constant (K_D_) of r*Pm*LGBP against r*Pm*FREP or r*Pm*FREP CC-MBP by varying analyte concentrations. The binding curves were globally fitted. The K_D_ values for r*Pm*FREP (Figure 6B) and the r*Pm*FREP CC-MBP (Figure 6C) interacting with r*Pm*LGBP were 0.34 μM and 0.22 μM, respectively. This finding demonstrates that *Pm*LGBP forms an immunological complex with *Pm*FREP through the CC regions at the N-terminus of *Pm*FREP.

## Discussion

PRRs play a crucial role in pathogen defense in the shrimp immune system. They recognize and bind to PAMPs and trigger immune responses. Hemocytes are key immune cells for the defense response of shrimp against pathogens. Several shrimp immunity processes occurred in hemocytes [40]. *Pm*FREP is a PRR that was first identified in the shrimp hemolymph [31]. *Pm*FREP serves as a PRR by recognizing N-acetyl sugar and peptidoglycan on bacterial pathogens such as *VP*_AHPND_ [30–32]. *PmFREP* transcripts were expressed in several shrimp tissues, including hemocytes [30]. Here, the expression of *PmFREP* in shrimp hemocytes upon *VP*_AHPND_ infection indicates the involvement of *Pm*FREP as a PRR during *VP*_AHPND_ infection. A previous study also showed that *PmFREP* transcripts in the shrimp stomach were up-regulated following *VP*_AHPND_ infection [33]. In addition, *Vibrio harveyi* infection induced the expression of *PmFREP* transcripts in shrimp hemocytes [30].

To observe the role of *Pm*FREP during *VP*_AHPND_ infection, *PmFREP* transcripts were suppressed. The *PmFREP* knockdown shrimp exhibited a reduced survival rate and increased susceptibility to pathogens compared with the *GFP* dsRNA-injected group. The *PmFREP* and *GFP* dsRNA-injected groups with no infection did not have a significant reduction in survival, suggesting that injection-related stress or contamination is not an issue in this experiment. The infected group exhibited 0% survival with a slight difference in time (120 vs 126 hours post-injection). This is expected because of the high dose of the *VP*_AHPND_ used in the experiment [41]. Still, with infection, the survival rate difference between the *PmFREP* and *GFP* dsRNA-injected groups appeared to be significant during 18–120 hpi, highlighting the role of *PmFREP* in shrimp immunity. Moreover, suppression of the *PmFREP* gene affected the hepatopancreas tissue damage after *VP*_AHPND_ infection, compared with the control group [33]. A previous study revealed that FREP from *F. merguiensis* (*Fm*LFd), which has 95% identity with *Pm*FREP, can promote immune processes like encapsulation and melanization [27]. The proPO activating cascade facilitates the production of melanin, a by-product that possesses antimicrobial properties. *Pm*LGBP acts as a PRR in the proPO pathway [42]. The binding between *Pm*LGBP and their PAMPs (LPS and β-1,3-glucan) activates the proPO enzyme to PO enzyme to catalyze melanin synthesis [43]. In our work, *PmFREP* knockdown shrimp exhibited lower expression of the *PmLGBP*, *PmproPO 1*, and *PmproPO 2* in the early stages of infection. This finding suggested that *Pm*FREP is possibly involved in melanin synthesis, similar to *Fm*LFd. In the insects *Manduca sexta* [44] and *Tenebrio molitor* [45], the serine proteases within the proPO cascade also participate in the Toll signaling pathway. This study revealed that *PmFREP* suppression could affect the transcription of genes associated with the Toll pathway. Interestingly, suppression of *PmFREP* not only affects the expression of proPO-related genes and Toll pathway genes but also affects the transcription of JAK/STAT signaling pathway-associated genes. In addition, *PmFREP* knockdown shrimp exhibited the reduction of antimicrobial peptide or protein gene expression (*PmPEN3* and *PmLysozyme C*) similar to the FREP from *Macrobrachium nipponense* [46]. Our results indicated that *PmFREP* plays an important role in shrimp immunity during *VP*_AHPND_ infection, thus *Pm*FREP serves as a PRR for the immune signaling pathway. The knockdown of *PmFREP* could lead to a delay in immune signaling, resulting in slower pathogen clearance and potentially increased susceptibility to pathogen infections.

In mammals, FREPs form complexes with other proteins. This ability is necessary for their participation in various biological processes, such as coagulation, cell signaling, and immune response. Human ficolins activate the complement system by forming a complex with mannose-binding lectin-associated serine proteases [47]. Moreover, FREPs from invertebrates like snails, *Biomphalaria glabrata*, can form complexes with TEP that participates in phagocytosis or encapsulation [22]. Although it is well documented that *Pm*FREP could bind to the pathogen, the signaling pathway after the binding event is still unclear. Here, we hypothesize that *Pm*FREP binds with other proteins to form an immunological complex. Therefore, we performed a pull-down assay to identify the binding partner and the immune signaling pathway that *Pm*FREP is involved in. Our discovery suggested that *Pm*LGBP is a binding partner of *Pm*FREP. However, the LC-MS/MS results indicated low coverage, likely due to the low protein yield, necessitating other supporting data [48]. The molecular weight of the *Pm*LGBP on SDS-PAGE was lower than its expected molecular weight, suggesting post-translational modification [38,42,43]. The *Pm*LGBP*–Pm*FREP interaction was confirmed by the curdlan binding assay. A previous study demonstrated that *Pm*LGBP binds with β-1,3-glucan [42], thus we employed curdlan as an affinity resin because it consists of β-1,3-linked glucose residues. As previously described, *Pm*LGBP serves as a PRR in the proPO cascade [42]. Since *Pm*FREP can interact with *Pm*LGBP, it could play a role in the proPO cascade. To verify this assumption, *PmFREP* expression level was suppressed, and PO activity was measured to examine the effect of *PmFREP* suppression on PO activity. *PmFREP* knockdown shrimp showed lower PO activity than control shrimp. As previously reported, PO activity of *PmLGBP* knockdown shrimp also decreased [42]. Despite the double knockdown, the reduction in PO activity was not significantly greater than that of the single knockdown. This could possibly be explained by the fact that the proPO activation pathway exhibits a degree of redundancy or compensatory mechanisms [43,49]. Even with the suppression of both *PmFREP* and *PmLGBP*, other receptors or activating factors may be capable of initiating the cascade [43]. Thus, the knockdown results on the PO activity may not be additive. The involvement of *Pm*FREP in the proPO cascade was further confirmed by investigating the effect of recombinant protein injection. Compared with the control shrimp, r*Pm*FREP-injected shrimp exhibited higher PO activity.

**Figure 7:**
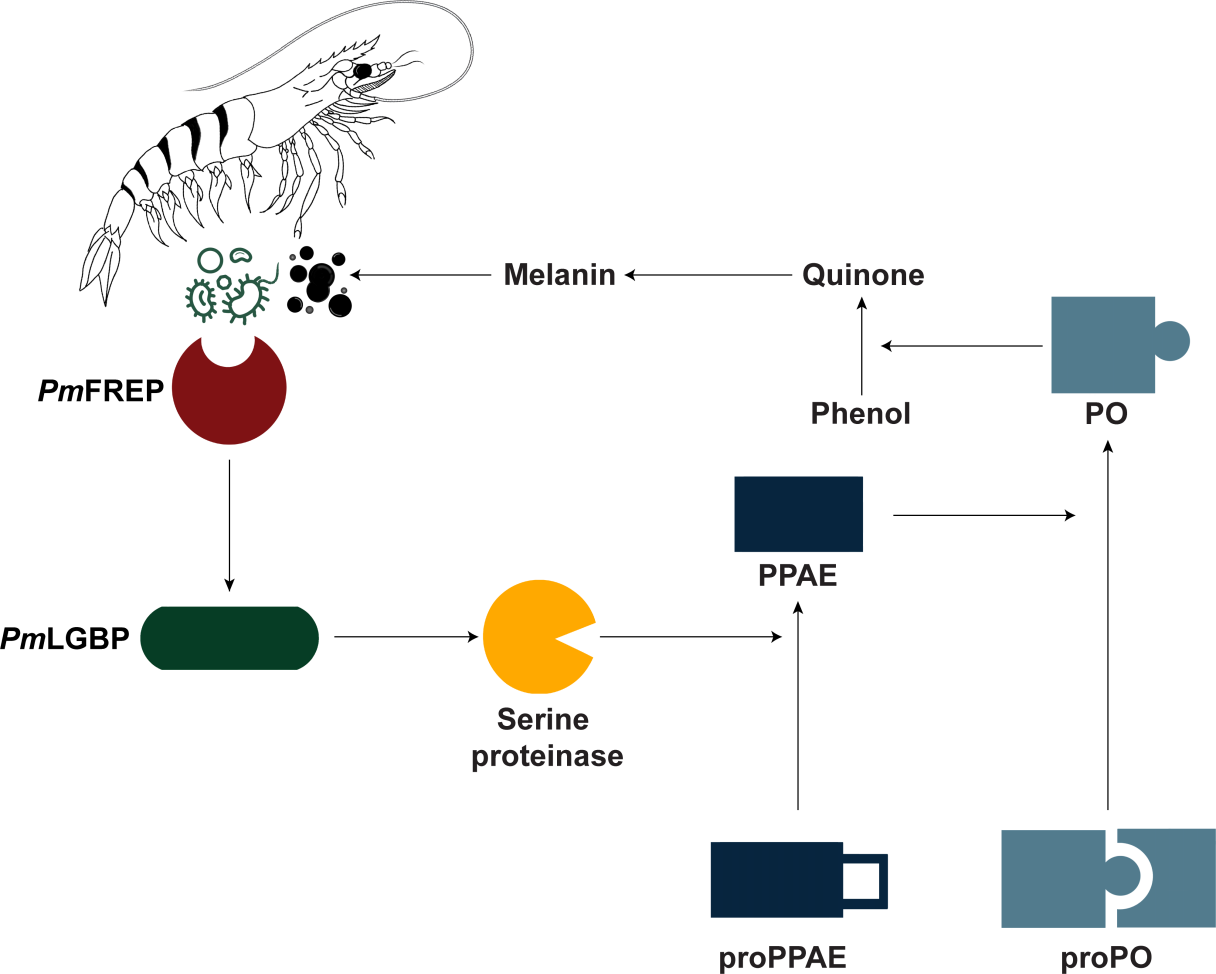
The proposed signaling pathway from the interaction between *Pm*FREP and the pathogen to melanization for pathogen inactivation.

*Pm*FREP comprises two CC regions at the N-terminus. CC regions of other lectins play an important role in biological recognition and signaling. For collectin liver 1 and collectin kidney 1, the CC region is crucial for the formation of trimers and higher-order oligomers, which increases the number of carbohydrate-binding sites available for interaction with pathogens [50,51]. The CC domains of a CTL from *M. japonicus* could bind to the domeless receptor and activate the JAK/STAT pathway to fight against bacterial infection [37]. Our results revealed increasing PO activity after r*Pm*FREP CC-MBP injection into shrimp during *VP*_AHPND_ infection; however, the PO activity in r*Pm*FREP CRD-injected shrimp was not different from the control group. Previous studies showed that r*Pm*FREP CRD is unable to bind with GlcNAc resin in a small spin column [32]. We increased the amount of GlcNAc resin from 100 µl to 8 ml to provide enough opportunity for protein interaction with the resin. Our investigation demonstrated that r*Pm*FREP CRD exhibited GlcNAc binding activity and hemagglutination. As expected, r*Li*DHFRL [39], a non-GlcNAc-binding protein, was unable to bind to the GlcNAc resin. Therefore, the r*Pm*FREP CRD cannot stimulate the PO activity, not due to incorrect folding but the lack of the CC region to form a complex with other proteins. We further confirm this assumption by verifying the binding between *Pm*LGBP and each domain of *Pm*FREP using a BLI assay, which is commonly used to study protein–protein interaction [52]. We found that only r*Pm*FREP and r*Pm*FREP CC-MBP could bind with r*Pm*LGBP. Our results demonstrated that *Pm*FREP is able to bind with *Pm*LGBP via the CC region. We speculate that the complex between *Pm*FREP and *Pm*LGBP likely activates the serine proteinase cascades that further catalyze proPPAE (pro proPO-activating enzyme) to PPAE (Figure 7). The PPAE further cleaves proPO to the active PO enzyme, resulting in the conversion of phenol to quinone that can self-polymerize to form melanin to surround the pathogen [43]. Future studies are necessary to support this hypothesis. The *Pm*FREP-activating proPO cascade resembles the lectin complement system activation in mammals. Both pathways involve activation of serine proteases by CC-containing lectins [53].

A previous study found that some insects have serine proteases that connect the proPO system and the Toll signaling pathway, which turns proSpätzle into Spätzle, acting as a signal for the toll receptor [45,54,55]. Our results show that *Pm*FREP suppression could affect Toll pathway transcripts as well. During melanin synthesis, the production of reactive oxygen species (ROS) occurs as a by-product. Peroxiredoxin 4 (Prx4) can detect this ROS and convert it to water [56]. Prx4 of the Kuruma shrimp is a ligand for Domeless in the JAK/STAT pathway [57]. Therefore, suppression of *Pm*FREP might affect the gene expression in the JAK/STAT pathway. However, our data only indicate changes at the level of gene expression. Additional experiments will be necessary to validate their functional connections.

In conclusion, *Pm*FREP plays a role in recognizing the N-acetyl sugar on the surface of the pathogen. We discovered that *Pm*FREP could form a complex with *Pm*LGBP, which further turns on the proPO system. The process led to melanin production for eliminating the pathogen. Activation of the proPO system by the *Pm*FREP*–Pm*LGBP complex is similar to the lectin complement pathway in mammals. These two pathways employ CC-containing lectins as receptors that signal through the serine proteinase cascades to eliminate the pathogen. Our study reveals a link between a CC-containing lectin and the innate immune signaling pathway in invertebrates and contributes to a broader understanding of lectin function.

## Methods

### Shrimp samples and ethics statement

Healthy shrimp, *P. monodon,* with an average body weight between 2–5 g, were purchased from a local farm in Chanthaburi province, Thailand. Shrimp were housed in the aquaculture facility in the Department of Biochemistry, Faculty of Science, Chulalongkorn University. Infection was carried out in a separate room from healthy animals. Shrimp were acclimated at least 14 days before the experiment in recirculating aerated seawater with 20 ppt salinity at an ambient temperature of 28 ± 1°C. They were fed with commercial food twice a day. Euthanasia was carried out by hypothermic shock in iced water. The experimental protocol was approved by the Animal Care and Use Committee of Faculty of Science, Chulalongkorn University (Protocol Review No. 2423015).

### Preparation of *VP*_AHPND_ immersion tank

The *V. parahaemolyticus* strain 5HP (*VP*_AHPND_) used was the previously isolated strain [58]. The bacteria were streaked onto Luria-Bertani (LB) agar containing 3% NaCl. To prepare a starter culture, a single colony of bacteria was inoculated into 50 ml of LB broth containing 3% NaCl and cultured at 30°C with 250 rpm shaking for 16 hours. The starter culture was then diluted at a 1:100 ratio into LB broth containing 3% NaCl and cultured at 30°C with 250 rpm shaking until the OD_600_ value reached 2 (10^8^ CFU per ml) [56]. The cells were collected by centrifugation at 3,000 × g for 5 minutes and then resuspended with 5 ml of sterile 0.85% w/v NaCl. The cell stock was then diluted into the seawater in an immersion tank at a final concentration of 5 × 10^5^ CFU/ml.

### Gene expression pattern of *PmFREP* after bacterial infection

To investigate the gene expression pattern, shrimp were immersed with *VP*_AHPND_ 5 × 10^5^ CFU/ml. After 0, 6, 24, and 48 hours of immersion, the hemocytes from 3 shrimp of each time point were collected. To prepare shrimp hemocytes, hemolymph was collected in a 1:1 ratio of an anticoagulant solution (30 mM tri-sodium citrate, 26 mM citric acid, 450 mM NaCl, 100 mM glucose, and 10 mM EDTA pH 7) [59] and centrifuged at 800 × g, 4°C for 10 minutes. The FavorPrep™ Tissue Total RNA Mini Kit (FAVORGEN, Taiwan) was used to extract the total RNA from shrimp hemocytes. The NanoDrop™ 2000 c Spectrophotometer (Thermo Fisher Scientific, USA) was used to measure the total RNA concentration. RNA (500 ng) was used for complementary DNA (cDNA) synthesis using Maxime™ RT PreMix (iNtRON Biotechnology). The RNA and cDNA were stored at -80°C.

The qRT-PCR was performed to quantify *PmFREP* transcript using Luna® Universal qPCR Master Mix (NEB, USA) by the CFX96 Touch™ Real-Time PCR Detection System (Bio-Rad, CA, USA). The synthesized cDNA, 20 ng, was used as a template with 0.2 µM gene-specific primers (Table 2) and 1 × Luna Universal qPCR Master Mix was added. Nuclease-free water was added to adjust the volume to 10 µl. The PCR reaction included one cycle at 95°C for 1 minute, followed by 40 cycles of 95°C for 15 seconds and 60°C for 30 seconds. After the quantification cycle (Cq) was measured, the relative expression was computed using Livak’s method (2^-^ **^ΔΔ^** ^Cq^). The *elongation factor 1α* (*EF1α*) gene was used as a reference gene. The ΔΔCq value was calculated as ΔCq of the infected group—ΔCq of the control group. The ΔCq value was computed from the Cq of *PmFREP* − Cq of *EF1α*. The experiment was performed with three replicates.

**Table 2:** The primers used for dsRNA synthesis and qPCR

Primers	Sequence	Purpose	GenBank accession number
q*Pm*FREP-F	GCTCAAAGTCGGCAGGTACAA	qRT-PCR	KF991000.1
q*Pm*FREP-R	CTCTCGTGCTCGCCTTCATAA	qRT-PCR	KF991000.1
q*Pm*LGBP-F	TCGACAACGATATCTGGGA	qRT-PCR	JN415536
q*Pm*LGBP-R	CCCGCGGCCGTTCATGCCCCAC	qRT-PCR	JN415536
q*Pm*proPO1-R	GGTCTTCCCCTCCCGCTTCG	qRT-PCR	AF099741
q*Pm*proPO1-F	GCCGCAGGTCCTTTGGCAGC	qRT-PCR	AF099741
q*Pm*proPO2-R	GCCAAGGGGAACGGGTGATG	qRT-PCR	FJ025814
q*Pm*proPO2-F	TCCCTCATGGCGGTCGAGGT	qRT-PCR	FJ025814
q*Pm*DOME-F	CTCAGGCTATGTTTCTCAGGATTCA	qRT-PCR	MW187497.1
q*Pm*DOME-R	CACGGCAGTTCCTTTATGGTCT	qRT-PCR	MW187497.1
q*Pm*STAT-F	TATATCCGAATGTGCCTAAG	qRT-PCR	EU367985.1
q*Pm*STAT-R	ATAGTTTGTGGTGTGTTGGG	qRT-PCR	EU367985.1
q*Pm*Lyzc-F	GCGGCAGCGATTATGGCAAG	qRT-PCR	GQ478702
q*Pm*Lyzc-R	TTGGAACCACGAGACCAGCACT	qRT-PCR	GQ478702
q*Pm*Toll-F	AGACCCTGCTCCTGGTGAGC	qRT-PCR	XM_037942098.1
q*Pm*Toll-R	TCCTGCCAGTGCCCCTTGAC	qRT-PCR	XM_037942098.1
q*Pm*Dorsal-F	TCACTGTTGACCCACCTTAC	qRT-PCR	MG775232.1
q*Pm*Dorsal-R	GGAAAGGGTCCACTCTAATC	qRT-PCR	MG775232.1
q*Pm*PEN3-F	GGCTTAGCCCCTTACA	qRT-PCR	FJ686016
q*Pm*PEN3-R	GACCCATACCTACAAATAAC	qRT-PCR	FJ686016
*EF1α*-F	AGGGCTTCGTAGCGTCGGTC	qRT-PCR	JN415536
*EF1α*-R	CGAAGGAACCTGTATTTGCT	qRT-PCR	JN415536
T7*Pm*FREP-F	GGATCCTAATACGACTCACTATAGGGGCCTGCAGGAGATGAGAAT	dsRNA synthesis	KF991000.1
T7*Pm*FREP-R	GGATCCTAATACGACTCACTATAGGAATGCCGGCCTTATCATCA	dsRNA synthesis	KF991000.1
T7*Pm*LGBP-F	GGATCCTAATACGACTCACTATAGGAGGGCTTCGTAGCGTCGGTC	dsRNA synthesis	JN415536
T7*Pm*LGBP-R	GGATCCTAATACGACTCACTATAGGCGAAGGAACCTGTATTTGCT	dsRNA synthesis	JN415536
T7GFP-F	GGATCCTAATACGACTCACTATAGGCAGTGCTTCAGCCGCTACCC	dsRNA synthesis	U55761
T7GFP-R	GGATCCTAATACGACTCACTATAGGAGTTCACCTTGATGCCGTTCTT	dsRNA synthesis	U55761
*Vp*TUMSAT-F	GTGTTGCATAATTTTGTGCA	qPCR	KP324996.1
*Vp*TUMSAT-R	TTGTACAGAAACCACGACTA	qPCR	KP324996.1

### Double-stranded RNA (dsRNA) synthesis

To understand the crucial role of *Pm*FREP during *VP*_AHPND_ infection, *PmFREP* was suppressed using the RNAi. *PmFREP* was amplified using primers containing the T7 promoter sequence in Table 2. The *GFP* dsRNA was synthesized using the pEGFP-1 vector (Clonetech) as a template. The PCR product was purified using the BioFact™ Gel & PCR Purification System (BIOFACT, Republic of Korea) and further used as a template for dsRNA synthesis. The dsRNAs were produced using the T7 RiboMAX™ Express Large Scale RNA Production System (Promega, USA). Briefly, 10 µl Ribomax™ Express T7 2X Buffer, 2 µl Enzyme Mix T7 Express, 1 µg T7 *PmFREP* PCR product, and nuclease-free water were mixed to obtain a total volume of 20 µl. After mixing, the component was incubated at 37°C for 30 minutes. To remove the DNA template, 1 µl RNase-free DNase was added and incubated at 37°C for 30 minutes. For annealing, the mixture was incubated at 70°C for 10 minutes and cooled down to room temperature for 20 minutes. To purify the dsRNA, 2 µl of 3 M CH_3_COONa and 20 µl of isopropanol were mixed with the component and incubated on ice for 5 minutes. To separate the precipitated dsRNA, the component was centrifuged at 18,000 x g for 10 minutes. The precipitated dsRNA was washed with cold 70% ethanol, centrifuged at 18,000 x g for 10 minutes, and dried for 30 minutes. Nuclease-free water (50 µl) was added to dissolve the dsRNA. The dsRNA concentration was measured using a NanoDrop™ 2000 c Spectrophotometer (Thermo Fisher Scientific, USA) and qualified using 2% w/v agarose-TAE gel electrophoresis.

### *In vivo* gene suppression of *PmFREP*

To suppress the *PmFREP* gene, shrimp with the body weight between 2–5 g were injected intramuscularly with 40 µl of *PmFREP* dsRNA or *GFP* dsRNA dissolved in sterile 0.85% w/v NaCl (5 µg/g shrimp) [33] at the third abdominal segment using a 0.5 ml insulin syringe. To quantify silencing efficiency, shrimp hemocyte was collected at 48 hours after injection, followed by RNA extraction and cDNA synthesis. The cDNA was subsequently used for qRT-PCR with the specific primers in Table 2. *EF1α* served as an internal control. The relative expression was determined using 2^-ΔΔCq^. The ΔΔCq value was calculated as ΔCq of *PmFREP*-silenced shrimp − ΔCq of *GFP* dsRNA-injected shrimp.

### Shrimp survival rate upon *VP*_AHPND_ infection

To study the effect of *PmFREP* suppression on shrimp survival rate upon *VP*_AHPND_ infection, healthy shrimp with 2–3 g body weight were divided into four groups of 30 shrimp per group. The four groups include *PmFREP* dsRNA-injected shrimp, *GFP* dsRNA-injected shrimp, *PmFREP* dsRNA-injected shrimp with *VP*_AHPND_ infection, and *GFP* dsRNA-injected shrimp with *VP*_AHPND_ infection. To suppress the *PmFREP* transcript, shrimp were injected with 5 μg dsRNA/g shrimp. The *GFP* dsRNA-injected shrimp served as the control group. At 48 hours after injection, shrimp were immersed in 5 × 10^5^ CFU/ml *VP*_AHPND_. Every 6 hpi, the numbers of dead shrimp were monitored. After 0, 48, and 120 hpi, the hepatopancreas were collected to count the *VP*_AHPND_ colony number on thiosulfate-citrate-bile salts-sucrose (TCBS) agar plates by serial dilution in sterile 0.85% NaCl. After incubating the plates at 30°C for 16 hours, the number of colonies was determined. The DNA was also extracted from the hepatopancreas of the dead shrimp using the FavorPrep™ Tissue Genomic DNA Extraction Kit (FAVORGEN, Taiwan). The qRT-PCR was performed to determine the *VP*_AHPND_ gene copy number using the DNA from hepatopancreas as a template with the *Pir* gene-specific primers (Table 2).

### Immune gene expression

To study the *PmFREP* signaling pathway, the relative expression of other immune genes was investigated. Three groups of immune-related genes explored included the proPO-associated gene (*PmLPBG*, *PmproPO1,* and *PmproPO2*), Toll pathway-related immune genes (*PmToll*, *PmDorsal,* and *PmPEN3*), and JAK/STAT pathway-related immune genes (*PmDome*, *PmSTAT,* and *PmLysozyme C*). At 48 hours after 5 µg dsRNA/g shrimp injection, shrimp were immersed in 5 × 10^5^ CFU/ml *VP*_AHPND_. At 3, 6, 12, and 24 hpi, the hemocytes of the shrimp were collected, followed by RNA extraction and cDNA synthesis. The relative expression of other immune genes was examined using qRT-PCR as previously described using specific gene primers (Table 2). The *GFP* dsRNA-injected shrimp served as a control group, and the *EF1α* gene expression served as an internal control. The relative expression was determined using 2^-ΔΔCq^. The ΔΔCq value was computed as the difference between ΔCq of *PmFREP*-silenced shrimp and ΔCq of *GFP*-injected shrimp.

### Recombinant protein expression and purification

To express r*Pm*FREP and r*Pm*FREP CRD by the insect cell expression system, the previously reported pFastBac1 XEEL SP His_6_ *Pm*FREP and pFastBac1 XEEL SP His_6_ *Pm*FREP CRD were used [32]. For r*Pm*LGBP production, pFastBac1 *Pm*LGBP was constructed by cloning the PCR product of the primers 5ʹ-GCGCGGATCCATGAAGGGCTTCGTAGCGTCGGTCG-3ʹ and 5ʹ-ATGCGGTACCTCATTAATGGTGATGGTGGTGATGCTGCTCGGTGCTCTCCATCTTCCAG-3ʹ into the BamHI and KpnI sites of pFastBac1. *P. monodon* hemocyte cDNA was used as the PCR template. The resulting r*Pm*LGBP has a C-terminal hexahistidine tag. The protein was produced using the Bac-to-Bac Baculovirus Expression System (Invitrogen, USA). The plasmids were transformed into the competent *E. coli* DH10BAC and selected using blue-white screening. Successful transposition in white colonies was confirmed by PCR using M13 primer. The protein was produced and purified as previously described [32].

To create the r*Pm*FREP CC-MBP and rMBP expression plasmids, the linker and the MBP gene (linker-MBP) from pMAL c5x (New England Biolabs, USA) was first amplified with the primers 5ʹ-AACAACAACAACAATAACAATAACAACAACAAAATCGAAGAAGGTAAACTGGTAATCTG-3ʹ and 5ʹ-GCACTCGAGCGAGCTCGAATTAGTCTGCGCGTC-3ʹ (primer R). This linker-MBP PCR product was then used as a template to construct both the r*Pm*FREP CC-MBP and the rMBP expression plasmids. The *Pm*FREP gene in pFastBac1 above was amplified by the primer 5ʹ- TATCCATGGGGACAACAGAACGAACAGATACCG -3ʹ (primer N) and the primer 5ʹ- GTTGTTGTTATTGTTATTGTTGTTGTTGTTGTGCCTCGGCCGCCGCCGCCGCCTC -3ʹ to yield the CC PCR product. To make the *Nco*I-r*Pm*FREP CC-MBP-*Xho*I DNA fragment, the CC and linker-MBP PCR products were mixed together and further amplified with the primers N and R. To make the *Nco*I-rMBP-*Xho*I DNA fragment, the linker-MBP PCR product was further amplified with the primers 5ʹ-TATCCATGGGGAACAACAACAACAATAACAATAACAACAACAAAATC-3ʹ and the primer R. Both gene fragments were cloned into the *Nco*I and *Xho*I sites of pET22b. The expression plasmids were verified by DNA sequencing. For protein expression, an overnight culture of the *E. coli* Tuner (DE3) harboring an expression plasmid was inoculated into 1 l of LB broth containing 100 μg/ml ampicillin. The cells were grown until OD_600_ ~ 0.6, and isopropyl β-D-1-thiogalactopyranoside (IPTG) was added to a final concentration of 1 mM. The cell was cultured at 16°C at 250 rpm for 16 hours. To obtain the protein expressed in the periplasmic space, osmotic shock was performed. After harvesting the cells by centrifugation, the cells were resuspended in 20 mM Tris-HCl, 20% sucrose, pH 8.0, and 1 mM EDTA, then incubated for 5–10 minutes at room temperature with shaking. The cells were collected by centrifugation at 8,000 x g for 20 minutes at 4°C. The pellet was resuspended in ice-cold 5 mM MgSO_4_ with stirring for 10 minutes on ice. The osmotic shock fluid was obtained as the supernatant after centrifugation at 8,000 x g for 20 minutes at 4°C. A stock solution of 1 M Tris-HCl, pH 7.5 was added to the osmotic shock fluid to achieve a final concentration of 20 mM Tris-HCl. The MBP fusion protein was subsequently purified using an amylose affinity chromatography by using 20 mM Tris-HCl pH 7.5 as the wash buffer and 20 mM Tris-HCl pH 7.5 with 10 mM maltose as the elution buffer. The purified fraction was dialyzed against 20 mM HEPES pH 7.5, 150 mM NaCl, and 25 mM imidazole for further purification with Ni-NTA affinity chromatography. The bound protein was eluted using the same buffer but supplemented with 250 mM imidazole. The protein was then dialyzed against the coupling buffer (20 mM HEPES pH 7.5, 150 mM NaCl, and 10 mM CaCl_2_) for storage. Protein purity was analyzed using SDS-PAGE. To determine protein concentration, spectrophotometry at 280 nm was used. The extinction coefficients of r*Pm*FREP, r*Pm*FREP CRD, r*Pm*FREP CC-MBP, rMBP, and r*Pm*LGBP are 63,745, 63,620, 67,840, 81,820, and 12,9370 M^-1^cm^-1^, respectively.

### Pull-down assay

*Pm*FREP was immobilized onto the Pierce™ NHS-Activated Magnetic Beads (Thermo Fisher Scientific, USA). Magnetic beads (50 µl) were first washed with ice-cold 1 mM hydrochloric acid. For protein coupling, 200 µl of 0.1 mg/ml r*Pm*FREP in the coupling buffer was added to the bead and incubated at room temperature for 2 hours on a rotator, followed by the bead collection using a magnet. The coupling step was done three times. The beads were then washed twice with 0.1 M glycine pH 2.0. The beads were quenched with 50 mM Tris pH 7.5, 150 mM NaCl, and 10 mM CaCl_₂_ for 3 hours. After quenching, the beads were washed with water and the coupling buffer, respectively. For the pulling down assay, shrimp hemolymph was collected from *VP*_AHPND_-infected shrimp using the anticoagulant solution and dialyzed against the coupling buffer using a 3K dialysis bag (Thermo Fisher Scientific, USA). Total hemolymph protein was determined using the Bradford protein assay (GoldBio, USA). Immobilized beads were incubated with 30 mg/ml of hemolymph and washed three times with 1 ml of the coupling buffer. The bound proteins were eluted using 2% SDS with boiling for 10 minutes. The supernatant was separated by centrifugation at 10,000 x g for 10 minutes. SDS-PAGE with silver staining was performed to analyze the eluted protein. As a control experiment, the quenched beads were used in the pull-down experiment. The protein band in the pull-down experiment that was different from the control experiment was cut and analyzed using LC-MS/MS (Ward Medic, Thailand). Briefly, the gel of the protein band was incubated in 25 mM NH₄HCO₃ at 4°C overnight and further reduced using 10 mM DTT at 56°C for 45 minutes. Alkylation was performed by adding 40 mM iodoacetamide and incubating in the dark at room temperature for 30 minutes. Trypsinization was then carried out using a trypsin ratio of 1:50 (w/w) at 37°C for 16 hours. To extract the peptide, 50% acetonitrile/0.1% formic acid was added, and the supernatant was aspirated. The peptide solution was then desalted using a Jupiter C18 column. The peptide sample was analyzed on a Q-Exactive™ Plus Hybrid Quadrupole-Orbitrap™ Mass Spectrometer with a 25 cm EASY-spray C18 column (75 µm diameter) connected to the EASY nano-LC 1000 instrument. Peptides were separated using an A-B gradient from 5% to 98% B over 90 minutes, at a flow of 300 nl/minute, where mobile phase A was 0.1% formic acid (Sigma, Cat No. 09676) in Pierce™ Water and mobile phase B was 0.1% formic acid in Pierce™ acetonitrile. Electrospray voltage was 2.0 kV. The resolution and scan range were 70,000 and 350–1,400 m/z, respectively.

### Curdlan binding assay

The protein (*rPm*FREP and/or *rPm*LGBP) at 0 or 0.1 µM each was incubated with 1 mg curdlan in the coupling buffer (125 µl) at 4°C for 16 hours on a rotator. After incubation, curdlan was collected by centrifugation at 10,000 x g for 5 minutes at room temperature and washed three times with 500 µl of 20 mM HEPES pH 7.5, 150 mM NaCl, and 10 mM CaCl_2_. The bound protein was eluted using 2% SDS with boiling for 10 minutes. Samples of all fractions were analyzed by Western blot with a monoclonal anti-His tag antibody (1:5000) (H1029, Sigma-Aldrich, USA). The Amersham ECL™ Prime Western Blotting Detection Reagents (Cytiva) and the Amersham ImageQuant 800 (Cytiva) were employed for western blotting signal detection. Three replicates of this experiment were conducted. The r*Pm*FREP band intensity was determined using ImageJ. The intensity of the r*Pm*FREP eluted fraction was normalized by the intensity of the r*Pm*FREP input fraction and multiplied by 100 to obtain the % intensity of the r*Pm*FREP eluted fraction from each experiment.

### Phenoloxidase (PO) activity assay

PO activity was carried out as previously described [60]. Briefly, 250 µg of total hemolymph protein was mixed with 25 µl of 3 mg/ml of 3,4-dihydroxy-L-phenylalanine (L-DOPA) (Sigma-Aldrich, USA). Tris pH 8.0 (10 mM) was added to obtain a total reaction volume of 100 µl. Melanin was measured at A_490_ for 1 hour. The PO activity was reported as ΔA_490_/60 minutes/total protein. For the knockdown experiments, RNAi was performed as described earlier. For the recombinant protein injection experiment, each recombinant protein was injected into shrimp at 1 nmol protein/g shrimp. At 24 hours after injection, shrimp were immersed in 5 × 10^5^ CFU/ml *VP*_AHPND_. The control group received rMBP injection.

### Hemagglutination and N-acetylglucosamine binding assays

The hemagglutination experiment was performed as previously published [61]. Briefly, a 2.5% suspension of porcine red blood cells in the binding buffer was incubated with r*Pm*FREP or r*Pm*FREP CRD at various concentrations in a 72-well agglutination plate (Terasaki plate) for 1 hour. For the GlcNAc binding assay, the GlcNAc resin was prepared as previously described [62]. This experiment was performed as previously reported with modifications [32]. The mixture r*Pm*FREP, r*Pm*FREP CRD, and r*Li*DHFRL (5 µM each) was applied to an 8 ml GlcNAc resin in an Econo-Pac® Chromatography column that was equilibrated with the binding buffer. The resin was washed with the binding buffer and eluted with the binding buffer supplemented with 100 mM GlcNAc. One-milliliter fractions were collected during the purification process. The fractions were analyzed by Western blot analysis using the monoclonal anti-His tag antibody described above. The r*Li*DHFRL served as a non-binding control [39].

### Biolayer interferometry (BLI)

BLI was performed using BLItz (ForteBio). The r*Pm*LGBP was immobilized on the Octet® SA Biosensor (SARTORIUS, Germany). The biosensor was first rehydrated in distilled water for 10 minutes and activated using 20 mM *N*-ethyl-*N*´-(3-dimethylaminopropyl)carbodiimide (EDC) and 10 mM NHS in water. The r*Pm*LGBP solution (36.5 µM) in the coupling buffer was used for immobilization by dipping the biosensor into the solution four times for 3 minutes each. The immobilized biosensor was then quenched with 50 mM Tris pH 7.5, 150 mM NaCl, and 10 mM CaCl_₂_. For each experiment, the baseline was measured by dipping the biosensor in the coupling buffer for 1 minute, followed by dipping the biosensor into the analyte solution for 4 minutes to obtain an association curve. For dissociation, the biosensor was dipped in the coupling buffer for 4 minutes. The data was examined with the BLItz Pro software.

### Statistical analyses

The *PmFREP* expression pattern studies**,** the effect of *Pm*FREP silencing, and curdlan binding assays were analyzed using a t-test with a significant difference at *p <* 0.05. The effect of *Pm*FREP on PO activity was analyzed using one-way ANOVA with a significant difference at *p <* 0.05. The results were displayed as means ± standard deviations. The graphs of results were generated using Prism 8.0 (GraphPad).

## Supplementary material

online supplementary material 1.

## Data Availability

Materials and data are available from the corresponding author upon a reasonable request.

## Open Access

This article has been published open access under our Subscribe to Open programme, made possible through the support of our subscribing institutions, learn more here: https://portlandpress.com/pages/open_access_options_and_prices#conditional

## Abbreviations

AHPND: acute hepatopancreatic necrosis disease
BLI: biolayer interferometry
CC: coiled-coil
CFU: colony-forming units
CRD: carbohydrate recognition domain
CTL: C-type lectin
Cq: quantification cycle
EF1α: elongation factor 1α
FBG: fibrinogen-like
FREP: fibrinogen-related protein
GlcNAc: N-acetylglucosamine
LB: Luria-Bertani
LGBP: lipopolysaccharide and β-1,3-glucan binding protein
MBP: mannose-binding protein
PAMPs: pathogen-associated molecular patterns
PL5: Penlectin5
PO: Phenoloxidase
PRRs: pattern recognition receptors
*Pm*FREP: *Penaeus monodon* fibrinogen-related protein
*Pm*LGBP: *Penaeus monodon* lipopolysaccharide and β-1,3-glucan binding protein
Prx4: Peroxiredoxin 4
RNAi: RNA interference
ROS: reactive oxygen species
TEP: thioester-containing protein
*VP*_AHPND_: *Vibrio parahaemolyticus* AHPND-causing strain
dsRNA: double-stranded RNA
hpi: hours post-infection
proPO: prophenoloxidase
proPPAE: pro proPO-activating enzyme
rMBP: recombinant mannose-binding protein
r*Pm*FREP: recombinant *Penaeus monodon* fibrinogen-related protein
r*Pm*LGBP: recombinant *Penaeus monodon* lipopolysaccharide and β-1,3-glucan binding protein
