# Supplementary material for: Penaeus monodon fibrinogen-related lectin interacts with lipopolysaccharide and β-1,3-glucan binding protein to activate the innate immune system
Source: Biochem J. 2025 Dec 17;482(24):1877–95. doi: 10.1042/BCJ20253314 (PMC12751051; doi:10.1042/BCJ20253314)
Supplement: online supplementary material 1. [file bcj-482-24-BCJ20253314-s001.pdf]

**Supporting Information**

# ***Penaeus monodon*** **fibrinogen-related lectin interacts with the lipopolysaccharide and beta-1,3-glucan binding protein to activate the innate immune system**

Patcharin Wilasluck[1,2], Pongsakorn Sukonthamarn[1], Anchalee Tassanakajon[1], Kittikhun Wangkanont[1,2,*]

[1]Center of Excellence for Molecular Biology and Genomics of Shrimp, Department of Biochemistry, Faculty of Science, Chulalongkorn University, Bangkok 10330, Thailand

[2]Center of Excellence in Molecular Crop, Department of Biochemistry, Faculty of Science, Chulalongkorn University, Bangkok 10330, Thailand

*Corresponding author

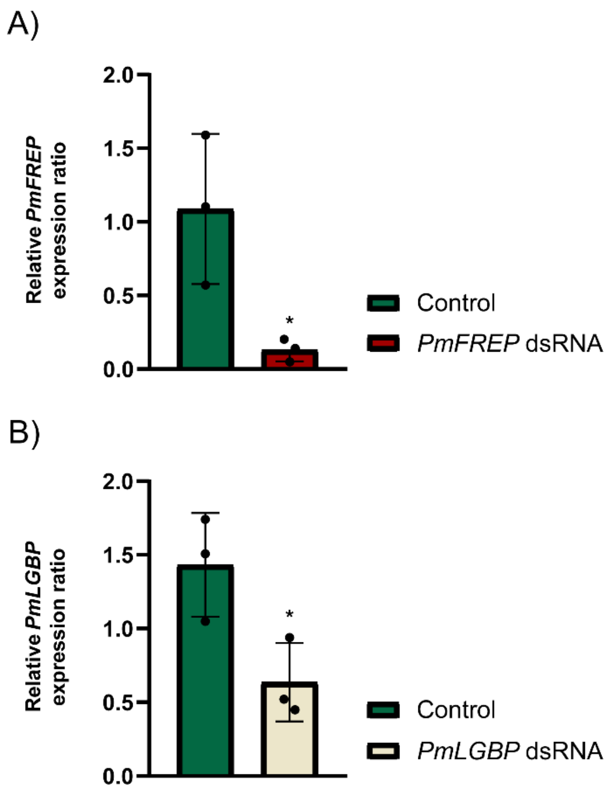


**Figure S1.** Gene suppression using RNA interference (RNAi). A) *PmFREP* transcripts were successfully suppressed using 5 µg dsRNA/g shrimp after 48 hours. B) *PmLGBP* transcripts were successfully suppressed using 5 µg dsRNA/g shrimp after 48 hours. *GFP* dsRNA-injected shrimps served as control experiments. The *PmEF1α tr*anscript levels were used as internal controls. The bar graph represents average relative expressions of three independent repeats ± 1 S.D. (error bars). A significant difference compared with the control was indicated as an asterisk ($p < 0.05$).

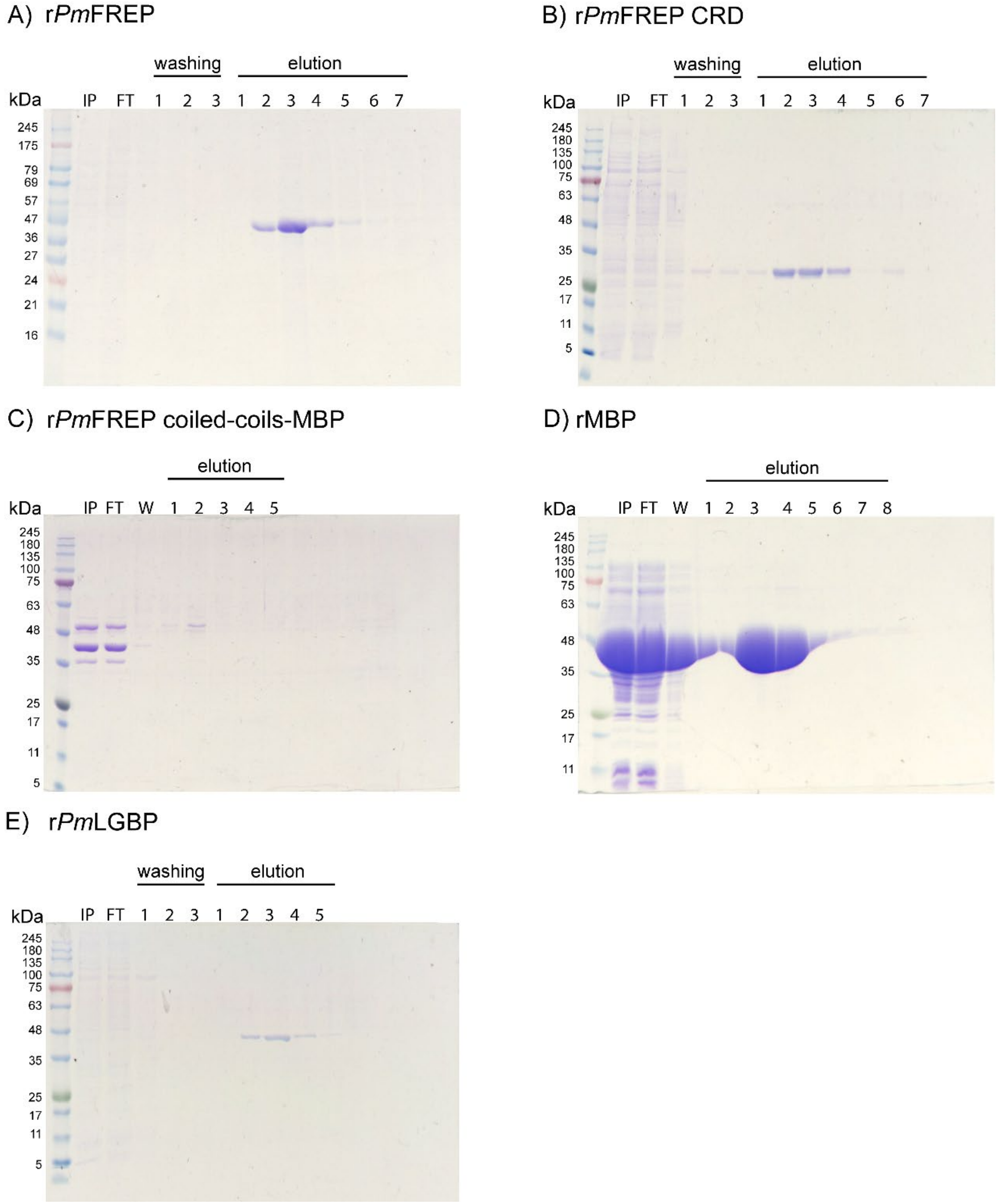


**Figure S2.** Recombinant protein purification using Ni-NTA affinity chromatography A) r*Pm*FREP. B) r*Pm*FREP CRD. C) r*Pm*FREP coiled coils-MBP. D) rMBP. E) r*Pm*LGB. IP = input. FT = flow-through. W or washing = sequential wash fractions. Elution = sequential elution fractions.

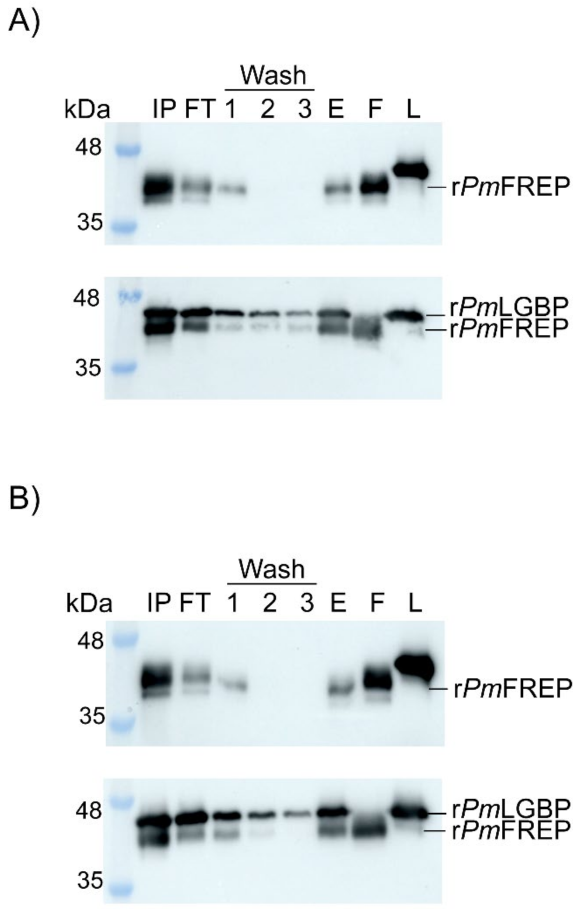


**Figure S3.** Binding of r*Pm*FREP and r*Pm*LGBP using a curdlan binding assay. A) Western blot analysis of the curdlan binding assay in the second replicate B) Western blot analysis of the curdlan binding assay in the third replicate. IP = input. FT = flow-through. Wash = sequential wash fractions. E = elution fraction. F = r*Pm*FREP. L = r*Pm*LGBP.

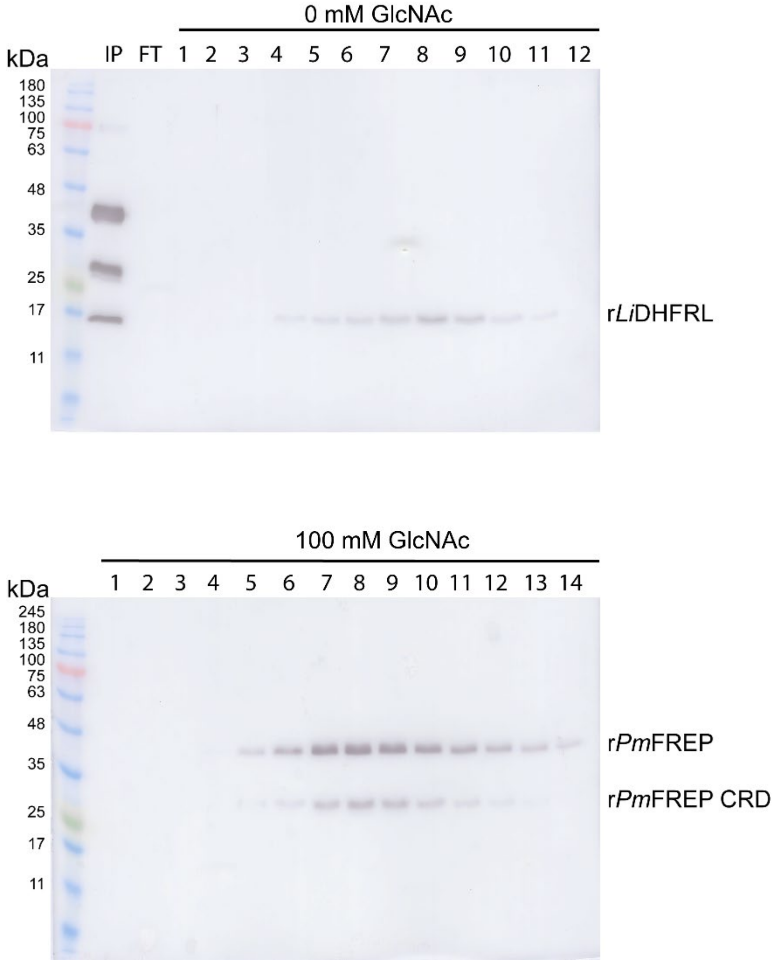


**Figure S4.** The GlcNAc binding activity of r*Pm*FREP CRD. r*Li*DHFRL served as a non-binding control. IP = input. FT = flow-through. The numbers indicate sequential elution fractions with the indicated GlcNAc concentrations.
